# Classification of Vogt-Koyanagi-Harada disease using feature selection and classification based on wide-field swept-source optical coherence tomography angiography

**DOI:** 10.3389/fbioe.2023.1086347

**Published:** 2023-05-02

**Authors:** Peng Xiao, Ke Ma, Xiaoyuan Ye, Gengyuan Wang, Zhengyu Duan, Yuancong Huang, Zhongzhou Luo, Xiaoqing Hu, Wei Chi, Jin Yuan

**Affiliations:** State Key Laboratory of Ophthalmology, Zhongshan Ophthalmic Center, Sun Yat-sen University, Guangzhou, China

**Keywords:** Vogt-Koyanagi-Harada (VKH), wide-field swept-source optical coherence tomography (WSS-OCTA), feature selection and classification, equilibrium optimizer (EO), support vector machine (SVM)

## Abstract

**Background:** Vogt-Koyanagi-Harada (VKH) disease is a common and easily blinded uveitis entity, with choroid being the main involved site. Classification of VKH disease and its different stages is crucial because they differ in clinical manifestations and therapeutic interventions. Wide-field swept-source optical coherence tomography angiography (WSS-OCTA) provides the advantages of non-invasiveness, large-field-of-view, high resolution, and ease of measuring and calculating choroid, offering the potential feasibility of simplified VKH classification assessment based on WSS-OCTA.

**Methods:** 15 healthy controls (HC), 13 acute-phase and 17 convalescent-phase VKH patients were included, undertaken WSS-OCTA examination with a scanning field of 15 × 9 mm^2^. 20 WSS-OCTA parameters were then extracted from WSS-OCTA images. To classify HC and VKH patients in acute and convalescent phases, two 2-class VKH datasets (HC and VKH) and two 3-class VKH datasets (HC, acute-phase VKH, and convalescent-phase VKH) were established by the WSS-OCTA parameters alone or in combination with best-corrected visual acuity (logMAR BCVA) and intraocular pressure (IOP), respectively. A new feature selection and classification method that combines an equilibrium optimizer and a support vector machine (called SVM-EO) was adopted to select classification-sensitive parameters among the massive datasets and to achieve outstanding classification performance. The interpretability of the VKH classification models was demonstrated based on SHapley Additive exPlanations (SHAP).

**Results:** Based on pure WSS-OCTA parameters, we achieved classification accuracies of 91.61% ± 12.17% and 86.69% ± 8.30% for 2- and 3-class VKH classification tasks. By combining the WSS-OCTA parameters and logMAR BCVA, we achieved better classification performance of 98.82% ± 2.63% and 96.16% ± 5.88%, respectively. Through SHAP analysis, we found that logMAR BCVA and vascular perfusion density (VPD) calculated from the whole field of view region in the choriocapillaris (whole FOV CC-VPD) were the most important features for VKH classification in our models.

**Conclusion:** We achieved excellent VKH classification performance based on a non-invasive WSS-OCTA examination, which provides the possibility for future clinical VKH classification with high sensitivity and specificity.

## 1 Introduction

Vogt-Koyanagi-Harada (VKH) disease is a T-cell-mediated multisystem autoimmune disease targeting melanocyte-containing organs (Ei Ei Lin Oo et al., 2020). The incidence of VKH varies among populations worldwide with a common occurrence in Hispanics (mestizos), Asians, Native Americans, Middle Easterners, and Asian Indians (Read et al., 2001). It causes not only ocular manifestations such as bilateral granulomatous panuveitis, chorioretinitis, and exudative retinal detachment (Ei Ei Lin Oo et al., 2020), but also multiple extraocular manifestations, including the central nervous system, auditory, and cutaneous abnormalities (O’Keefe and Rao, 2017). According to the multimodal ocular vascular imaging approach, VKH is clinically manifested in four phases, specifically prodromal, acute, convalescent, and chronic recurrent phases (Luo et al., 2021). The prodromal phase starts rapidly, often with cold symptoms, and develops into the acute phase quickly. Early diagnosis, timely initiation of treatment, and appropriate and adequate therapy are key to optimal VKH management, while delayed diagnosis and initiation of appropriate therapy may lead to an increased risk of disease chronicity, complications, and visual impairment (Ei Ei Lin Oo et al., 2020). For example, the acute phase, if not adequately treated, can progress to the chronic recurrent stage (Agarwal et al., 2020). In contrast, with appropriate treatment such as corticosteroids, the disease will transition to the convalescent phase after several weeks to months. Because of the different treatment regimens and dosages in the acute and convalescent phases, it is important to accurately identify and classify VKH, especially in these two phases, so that patients with VKH can be given the proper and suitable treatment to recover and avoid chronic symptoms.

At present, the classification of VKH mainly focuses on clinical manifestations, supplemented by auxiliary examinations such as fluorescein angiography (FA), indocyanine green angiography (ICGA), and optical coherence tomography (OCT) (Li et al., 2021). The FA and ICGA have long been used as validation tools for the classification and evaluation of VKH, however, both are time-consuming, invasive, and non-quantitative, and also have potential dye-related risks (Qian et al., 2021). The enhanced depth imaging OCT (EDI-OCT) and swept-source OCT (SS-OCT) are non-invasive, more affordable techniques for assessing choroidal thickness and morphology (Urzua et al., 2020). Moreover, it has been shown that SS-OCT is superior to EDI-OCT, providing higher resolution and more measurable images and accurate qualitative and quantitative assessment of retinal and choroidal changes (Chee et al., 2017). The SS-OCT angiography (SS-OCTA) provides not only the advantages of SS-OCT such as non-invasive and high resolution but also the advantages of tissue penetration, visualizing and quantitatively measuring the retinal and choroidal vascular system with high repeatability and reproducibility, making it increasingly important and widely used in the field of uveitis (Liang et al., 2021). Several studies have used SS-OCTA to demonstrate choroidal retinal microvascular changes in VKH disease (Karaca et al., 2020; Liang et al., 2020; Fan et al., 2021). Compared with SS-OCTA, wide-field SS-OCTA (WSS-OCTA) can obtain a larger field of view and extract more vascular features, and thus is gradually being applied in VKH studies (Qian et al., 2021; Ye et al., 2021). However, to the best of our knowledge, there is no classification study or criteria for VKH based on the WSS-OCTA.

In addition, WSS-OCTA can extract many features, such as up to 20 features in our previous work (Ye et al., 2021), which help to analyze microvascular alterations in VKH patients. However, for VKH classification, high-dimensional features like these may contain not only relevant features but also irrelevant and redundant features, which can reduce VKH classification performance. Feature selection is one of the effective ways to reduce dimensionality, which helps to reduce the risk of overfitting, improve the generalization ability of the model and save computational effort because only a fewer features are calculated (Shilaskar and Ghatol, 2013). On the other hand, machine learning methods, such as support vector machines (SVM), logistic regression (LR), random forests (RF), K-nearest neighbors (KNN), and decision trees (DT), are widely used for classification and prediction of ophthalmic diseases, such as myopia and keratitis (Tang et al., 2020; Herber et al., 2021), glaucoma, uveitis, cataract, and age-related macular degeneration (Lin et al., 2020; Standardization of Uveitis Nomenclature SUN Working Group, 2021b; Ting et al., 2021), and recently also for VKH classification (Standardization of Uveitis Nomenclature SUN Working Group, 2021a; Chang et al., 2021), because of their good classification performance in small datasets. These classifiers are often trained with hyperparameters, which need to be optimized to obtain the best classification performance. Therefore, considering these two aspects, this paper attempts to achieve accurate VKH classification based on a small number of WSS-OCTA features by simultaneously performing the selection of numerous WSS-OCTA features, hyperparameter optimization of the classifier, and VKH classification. In short, this paper aims to investigate a simplified and accurate VKH classification method based on WSS-OCTA unimodal imaging. To achieve this, this paper proposes a new feature selection and

Classification method by combining an equilibrium optimizer (EO), a metaheuristic algorithm with strong search power (Faramarzi et al., 2020), and an SVM, named SVM-EO, to build accurate two-class (healthy control and VKH) and three-class (healthy control, acute-phase, and convalescent-phase VKH) VKH classification models. To verify the feasibility and validity of the method, two feature combination schemes were tested in this paper, namely, pure WSS-OCTA features and combined two basic clinical characteristics and WSS-OCTA features.

## 2 Methods

### 2.1 Subjects

This single-center study was conducted at the Zhongshan Eye Center in China and in accordance with the Declaration of Helsinki, and all participants signed informed consents. Table 1 gives a detailed information of the patients included, which was reported in our previous publication (Ye et al., 2021). The classification of VKH disease was made by experienced ophthalmologists in strict accordance with the revised classification criteria (RDC) developed by the First International Workshop on Vogt-Koyanagi-Harada (VKH) disease (Read et al., 2001). The acute VKH group and the convalescent VKH group were divided according to disease progression. For example, inclusion criteria for the acute VKH group were patients who were initially diagnosed or treated with systemic corticosteroids for less than 2 weeks and excluded severe exudative retinal detachment, severe anterior chamber inflammation, or vitreous opacities, whereas patients with convalescent VKH were those who had been treated with systemic corticosteroids for more than 3 months and did not have acute fundus inflammation. The healthy controls were included in the absence of ocular or systemic disease and matched for age and number of cases to the VKH group. In addition, some ophthalmic examinations were performed for all participants, including the logarithm of the minimum angle of resolution of best corrected visual acuity (BCVA) measured with a Snellen chart (logMAR BCVA), bilateral intraocular pressure (IOP), slit lamp microscopy, indirect fundus ophthalmoscopy, and FA examinations.

**TABLE 1:** The detailed information of the patients included (Ye et al., 2021).

	Healthy controls	Acute-phase VKH	Convalescent phase VKH	*p*-value
Number of eyes/patients	30/15	20/13	30/17	
Age (years)	34 (26–58)	34 (27–53)	36 (29–43)	0.971^a^
Sex (male/female)	5/10	8/5	9/8	0.302^b^

Notes: Patients’ ages are presented as Median (P25-P75).

^a^ Kruskal–Wallis test.

^b^ Chi-square test.

### 2.2 WSS-OCTA image acquisition and feature extraction

**FIGURE 1:**
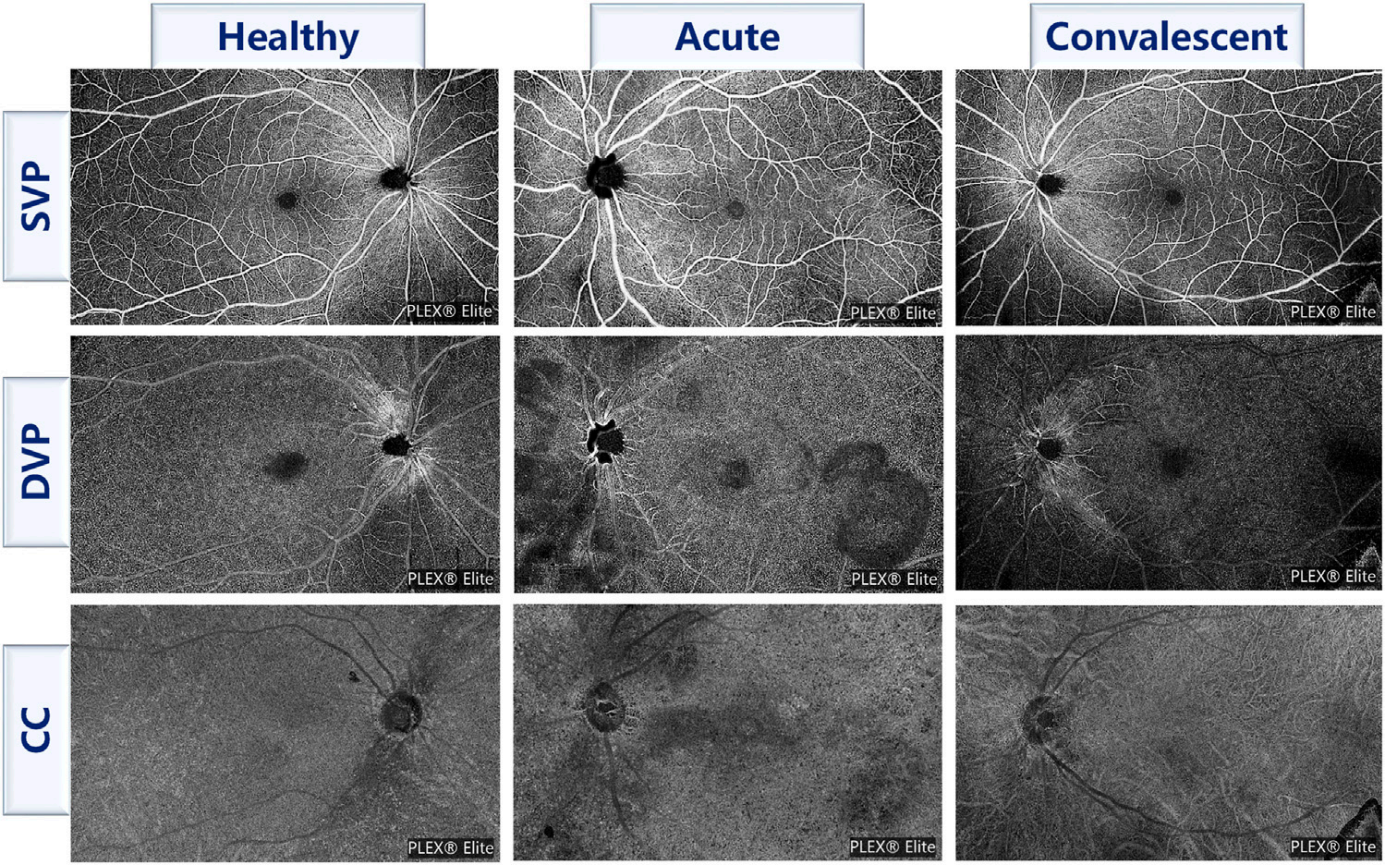
Examples of three slabs for three classes of participants. SVP, superficial vascular plexus; DVP, deep vascular plexus; CC, choriocapillaris.

All WSS-OCTA images were acquired by an experienced technician using the PLEX Elite 9000 device (Carl Zeiss Meditec Inc., Dublin, CA, united states of america) with a central wavelength of 1,060 nm and a speed of 100,000 A-scans per second to perform a 15 × 9 mm^2^ scan centered on the fovea of each eye. The PLEX Elite 9000 device has an active eye-tracking system with its auto-focus function to minimize the effects of eye aberrations when taking images. Poor quality WSS-OCTA images with a lot of motion artifacts or incorrect segmentation were excluded. All WSS-OCTA images were automatically segmented using the built-in software to generate superficial vascular plexus (SVP), deep vascular plexus (DVP), and choriocapillaris (CC), as shown in Figure 1 for an example. Then, four kinds of features were extracted from three slabs, namely, foveal avascular zone (FAZ), vascular perfusion density (VPD), vascular length density (VLD), and flow void (FV) across regions and layers parameters. The FAZ features included the area of the FAZ (AFAZ) and the acyclicity index (AI, the ratio of the FAZ perimeter to the perimeter of a circle of equal area) (Karaca et al., 2020) in SVP. To calculate the VPD, the ratio of vascular area to the total region of interest (ROI) (Triolo et al., 2017), 7 ROIs were selected, including the macular region in SVP, DVP, and CC, the peripapillary region in SVP, and the whole field of view (FOV) region in SVP, DVP, and CC, and thus a total of 7 VPD features were calculated, namely, macular SVP-VPD, macular DVP-VPD, macular CC-VPD, peripapillary SVP-VPD, whole FOV SVP-VPD, whole FOV DVP-VPD, and whole FOV CC-VPD. VLD is the length of vessels per unit area (Uji et al., 2017), allowing for a more sensitive classification of small blood vessels and capillaries. Similarly, 5 ROIs were first selected, namely, the macular region in SVP and DVP, the peripapillary region in SVP, and the whole FOV in SVP and DVP, and finally 5 VLD features were obtained, including macular SVP-VLD, macular DVP-VLD, peripapillary SVP-VLD, whole FOV SVP-VLD, and whole FOV DVP-VLD. The FV in CC was determined by thresholding the areas lacking flow information (Spaide, 2016). FV variables, including FV area ratio (FVAR), the number of FV areas larger than 1,000 um^2^ (FV1000), and the average size of the FV (FVAS), were calculated separately for the macular region and the peripheral region in CC, so altogether six FV features were extracted, which were macular CC-FVAR, macular CC-FV1000, macular CC-FVAS, peripheral CC-FVAR, peripheral CC-FV1000, and peripheral CC-FVAS. In brief, 20 WSS-OCTA features were extracted, including 2 FAZ features and 18 vascular features. A detailed description of the image processing including removing artifacts and extraction of OCTA parameters was given in our previous work (Ye et al., 2021). In addition, each feature was standardized for eliminating the effects of different feature magnitudes and accelerating convergence.

### 2.3 Feature selection and classification

**FIGURE 2:**
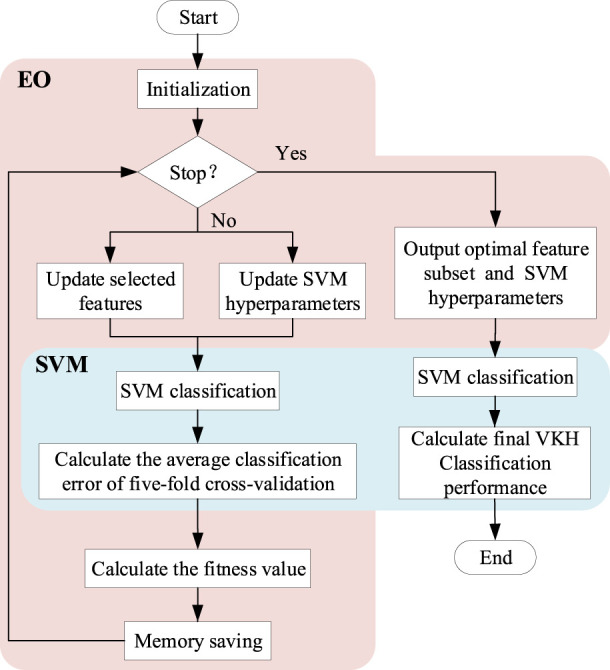
Flow chart of the feature selection and classification method combining EO and SVM. EO, equilibrium optimizer; SVM, support vector machine; VKH, vogt-koyanagi-harada.

To classify VKH disease accurately and objectively using as few parameters as possible, we adopted a feature selection and classification method combining EO and SVM (Figure 2), called SVM-EO, to achieve feature selection, SVM hyperparameter optimization, and classification simultaneously, which were implemented in MATLAB 2020b (MathWorks Inc., Natick, MA, United States). EO was used to update the feature subset and SVM hyperparameters, and SVM was applied to classification and construct the fitness function for EO.

#### 2.3.1 Equilibrium optimizer

Faramarzi et al. (2020) proposed EO inspired by the control volume mass balance model used to estimate the dynamic and equilibrium states. The mass balance equation is generally described using a first-order differential equation, and is calculated as:

$$C=C_{eq}+\left(C_{0}-C_{eq}\right)F+\frac{G}{\gamma V}\left(1-F\right)$$ (1)

where V is the control volume, C is the concentration inside the control volume, $C_{eq}$ is the concentration at equilibrium, G is the mass generation rate inside the control volume, F is the exponential term coefficient, γ is the flow rate, and $C_{0}$ is the initial concentration of the control volume.

The EO is mainly based on Eq. 1 for iterative optimization searching, where C represents the newly generated current solution, $C_{0}$ represents the solution obtained in the previous iteration, and $C_{eq}$ represents the best solution currently found by the algorithm. The implementation process of EO is briefly described as follows. First, each variable requiring optimization is initialized randomly based on the upper and lower bounds. Then EO enters the main iteration process to update the solution and memory saving. Specifically, the equilibrium state pool $C_{eq}$ consisting of five candidate solutions is constructed to improve the global search capability of the algorithm and avoid getting trapped in low-quality local optimal solutions. The coefficient F is updated for better balancing the global search and local search and the mass generation rate G is calculated to enhance the local search capability, see Eqs 2, 3. Thus, the current solution C is updated based on Eq. 4. Next, the fitness value of each member of the current solution is compared with that of the previous iteration and covered if it has a better fitness value to achieve memory saving. The above iterative process is repeated until the iteration ends and all optimal variables are output.

$$\left\{\begin{array}{c} F=a_{1}sign\left(r-0.5\right)\left[e^{-\gamma t}-1\right]\\ t={\left(1-\frac{Ite}{{Ite}_{\max}}\right)}^{a_{2}\frac{Ite}{{Ite}_{\max}}} \end{array}\right.$$ (2)

$$G=\left\{\begin{array}{c} 0.5\gamma_{1}\left(C_{eq}-\gamma C\right)F,\gamma_{2}\geq GP\\ 0,\gamma_{2}<GP \end{array}\right.$$ (3)

$$C=C_{eq}+\left(C-C_{eq}\right)F+\frac{G}{\gamma V}\left(1-F\right)$$ (4)

where $a_{1}$ and $a_{2}$ are constant, γ is a random vector between [0,1], Ite and ${Ite}_{\max}$ are the number of current iterations and total iterations, $\gamma_{1}$ and $\gamma_{2}$ are random numbers between [0,1], and GP is the generation probability.

#### 2.3.2 Support vector machine

SVM is a binary linear classifier that tries to minimize structural risk (Ma et al., 2019). It classifies two classes of samples by constructing the optimal hyperplane to maximize the sample intervals, which is a convex quadratic programming problem and the final mathematical model is:

$$\left\{\begin{array}{c} \min\frac{1}{2}{\left\|W\right\|}^{2}+\alpha \sum_{i=1}^{M}\varepsilon_{i}\\ s.t.{lab}_{i}\left(W^{T}{feat}_{i}+b\right)\geq 1-\varepsilon_{i}\\ \varepsilon_{i}\geq 0 \end{array}\right.$$ (5)

where α is the penalty factor, ε is the slack variable, M is the number of training samples, $\left({feat}_{i},{lab}_{i}\right)$ is the i th sample, and $\left(W,b\right)$ is the hyperplane parameter. To build a three-class VKH classification model, the one-versus-one method was adopted for the SVM to achieve multi-classification. In addition, the radial basis function (RBF) kernel was used to achieve nonlinear classification because it has only one hyperparameter, bandwidth β , and has been shown to perform well (Caramia et al., 2018). Therefore, to establish accurate SVM classification models for diagnosing VKH, the penalty factor α and bandwidth β were simultaneously optimized.

#### 2.3.3 Combined EO and SVM to classify VKH

The combination of EO and SVM maximizes the powerful search capability of EO and the excellent classification performance of SVM to efficiently detect VKH. First, initialize the input constants ( V=1 ; $a_{1}=2$ ; $a_{2}=1$ ; ${Ite}_{\max}=100$ ,; GP=0.5 ) and the parameters for optimization as:

$$C_{i,j}^{0}=\left\{\begin{array}{c} \alpha_{l}+\gamma_{j}\left(\alpha_{u}-\alpha_{l}\right),j=1\\ \beta_{l}+\gamma_{j}\left(\beta_{u}-\beta_{l}\right),j=2\\ F_{l}+\gamma_{j}\left(F_{u}-F_{l}\right),j>2 \end{array}\right.$$ (6)

where $C_{i,j}^{0}$ represents the initial value of the j th ( j=1,2,3,…,J ) dimension of the i th ( i=1,2,3,…,I ) member of the population, I and J are the number of population members and the sum of the number of features and SVM hyperparameters, $\gamma_{j}$ is a random number between [0, 1], and $\left.{\left[\alpha \right.}_{l},\alpha_{u}\right]$ , $\left.{\left[\beta \right.}_{l},\beta_{u}\right]$ , and $\left.{\left[F\right.}_{l},F_{u}\right]$ represent the upper and lower bounds of α , β , and features and take the values of [0.001,1000], [0.001,1000], and [0,1]. Then, if the number of the current iteration is less than the total number, the main iteration process is performed, whereupon the selected features and two SVM hyperparameters are updated. Notably, for the current feature $C_{i,j}\;\left(j>2\right)$ , if the calculated $C_{i,j}$ is greater than 0.5, then $C_{i,j}$ is taken as 0, indicating that this feature is discarded. Conversely, $C_{i,j}$ is 1, meaning that the feature is selected and all features corresponding to $C_{i,j}=1$ consist of the current feature subset.

Further, VKH classification was performed using SVM with updated hyperparameters based on the current feature subset, whose classification performance was stably evaluated using the five-fold cross-validation method. In detail, each class of samples in this feature subset was randomly divided into 5 folds according to the participants approximately equally, respectively, and then each fold of all classes was combined separately to form a 1-fold data subset containing all classes. Each 4-fold data subset was used to train the SVM model, and the remaining 1-fold was used to test the trained SVM model. And the average classification error $\bar{{Error}_{row}}$ obtained after 5 iterations was applied to construct the fitness function as:

$${Fitness}_{i}=\varphi \bar{{Error}_{i}}+\left(1-\varphi \right)\frac{{Num}_{i}}{J-2}$$ (7)

where ${Fitness}_{i}$ is the fitness value of the i th member of the population, φ is the coefficient that balances the classification performance and the number of features ( φ=0.98 ), and ${Num}_{i}$ is the number of the current feature subset. After that, the fitness value of each member of the current solution (population) was compared with that of the previous iteration for memory saving. After the main iteration, the best feature subset and the optimized SVM hyperparameters were output and used for SVM classification. To comprehensively assess the final VKH classification performance, sensitivity, specificity, accuracy, the receiver operating characteristic (ROC) curve and the area under the curve (AUC) were used as evaluation metrics and classification results based on five-fold cross-validation were given in the form of mean ± standard deviation (SD).

### 2.4 Global and local interpretability of the VKH classification models

Although the optimal feature subset and the best classification performance could be obtained by the SVM-EO method, the corresponding 2-class and 3-class VKH classification models are black-box models and lack interpretability. SHapley Additive exPlanations (SHAP) is a Shapley value-inspired additivity explanatory model based on game theory (Lundberg and Lee, 2017). SHAP assigns an importance value to each feature for a particular prediction, quantifying the magnitude and direction (positive or negative) of the feature’s influence on the prediction, so it is possible to interpret the outputs of any machine learning model. And its interpretable performance has been widely proven and applied (Wang et al., 2021; Baptista et al., 2022; Nohara et al., 2022; Onsree et al., 2022). Therefore, we used the SHAP package in Python to achieve global and local interpretability of the best VKH classification models. Specifically, the classification model that achieved the best classification performance in the five-fold cross-validation was interpreted, and we used the kernel explainer in the SHAP package because it is model-independent and works with any model. The SHAP summary plot (Lundberg et al., 2020) was applied for global interpretability, displaying the feature importance of all features. The longer the bar corresponds to each feature, the more important this feature is. The SHAP force plot (Lundberg et al., 2018) was used to achieve local interpretability, showing how the prediction of a single sample was generated. The combined effect of all features pushed the model’s predictions from the base value (the average prediction across all samples) to the final model output (f(x), the predicted value in this example). The features that pushed the prediction up and down were shown in red and blue respectively, and the wider the color area, the greater the impact of the feature.

### 2.5 Comparison and statistical analysis

We compared the classification performance based on pure WSS-OCTA features and based on clinical characteristics and WSS-OCTA features for two- and three-class VKH diagnoses, respectively. We ran the proposed SVM-EO method 30 times and considered the best classification performance as the final VKH classification performance. The statistical analysis was conducted in SPSS 24 (SPSS Inc., Chicago, IL, United States). The Mann-Whitney U test was performed to assess differences in classification performance due to different features and classes. Statistical significance was set at *p* ≤ 0.05.

## 3 Results

### 3.1 Dataset

In this study, 20 eyes of 13 VKH patients in the acute phase, 30 eyes of 17 VKH patients in the convalescent phase, and 30 eyes of 15 age-matched healthy controls were included and analyzed. Four datasets were formed based on 2 clinical characteristics (IOP and logMAR BCVA) and 20 WSS-OCTA features for building two- and three-class VKH classification models, as follows: Dataset1: a two-class dataset consisting of 20 WSS-OCTA features; Dataset2: a two-class dataset composed of all 22 features; Dataset3: a three-class dataset made up of 20 WSS-OCTA features; Dataset4: a three-class dataset comprising all 22 features.

### 3.2 Two-class VKH classification results based on Dataset1 and Dataset2

**FIGURE 3:**
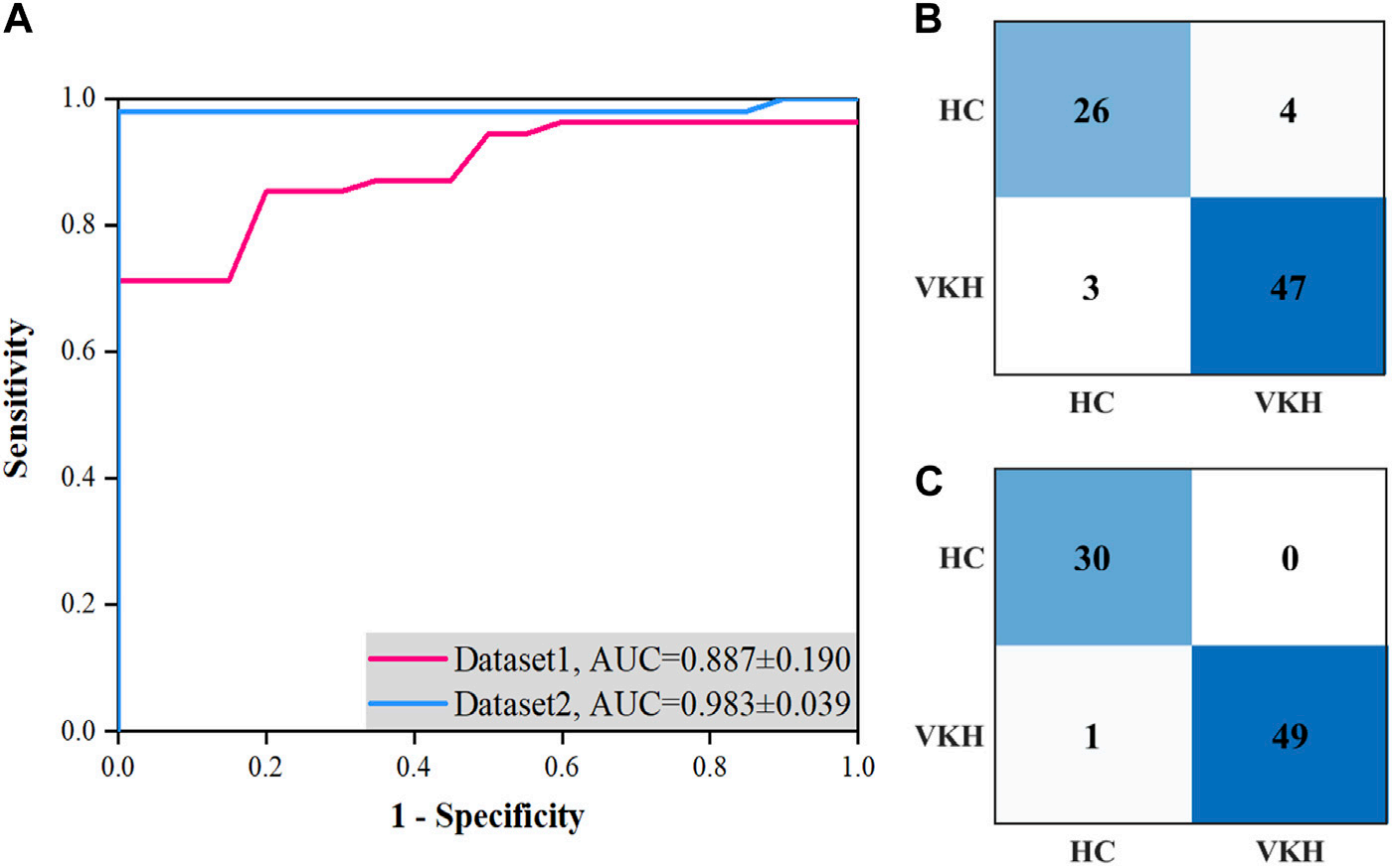
Best ROC curves and confusion matrices for two-class VKH classification with the SVM-EO method based on Dataset1 and Dataset2 using the five-fold cross-validation. **(A)** Best ROC curves. **(B)** Confusion matrix based on Dataset1. **(C)** Confusion matrix based on Dataset2.

We compared the average and best two-class VKH classification results obtained by the SVM-EO method based on Dataset1 and Dataset2. According to Table 2, the sensitivity, specificity, and accuracy obtained based on Dataset2 were significantly better than those acquired using Dataset1 concerning the average results. In terms of the best results, the sensitivity, specificity, and accuracy obtained based on Dataset2 were also superior to those gained with dataset1, although there was no significant difference, a smaller standard deviation was achieved. The best AUC obtained using Dataset2 was also markedly higher than that achieved by Dataset1 (Figure 3A), although there was no significant difference (*p* = 0.421). There are 7 eyes misidentified in Dataset1 (Figure 3B), while only one eye is misidentified in Dataset2 (Figure 3C). In terms of the features selected, the best feature subset obtained with Dataset1 contained 5 features, which were AFAZ, whole FOV DVP-VLD, macular SVP-VPD, peripheral CC-FVAR, and macular CC-FV1000, while the best feature subset gained from Dataset2 consisted of 4 features, namely, logMAR BCVA, macular DVP-VLD, whole FOV SVP-VPD, and whole FOV CC-VPD.

**TABLE 2:** Average and best two-class VKH classification results obtained by using the proposed SVM-EO method.

Dataset	Sensitivity (%)		Specificity (%)		Accuracy (%)	
	Average	Best	Average	Best	Average	Best
Dataset1	89.30 ± 5.62	94.36 ± 8.24	82.27 ± 7.06	86.67 ± 21.73	86.66 ± 5.40	91.61 ± 12.17
Dataset2	95.60 ± 1.62	98.00 ± 4.47	99.46 ± 2.96	100.0 ± 0.0	97.19 ± 2.09	98.82 ± 2.63
*P*	<0.001	0.548	<0.001	0.310	<0.001	0.222

### 3.3 Three-class VKH classification results based on Dataset3 and Dataset4

For the average classification performance, the sensitivity and specificity of the HC, acute-phase VKH, and convalescent-phase VKH classes gained from Dataset4 were significantly outperformed by those.

Obtained with Dataset3 (*p* < 0.001, Table 3). The average overall accuracy obtained using Dataset4 was also significantly more favorable than that achieved with Dataset3 (*p* < 0.001). From the best three-classification results, the sensitivity of the HC class and the specificity of the convalescent-phase VKH class based on Dataset4 were significantly greater than those of Dataset3 (*p* < 0.05). Unexpectedly, the.

**TABLE 3:** Average and best three-class VKH classification results obtained by using the feature selection and classification method.

Dataset	Class	Sensitivity (%)		Specificity (%)		Overall accuracy (%)	
		Average	Best	Average	Best	Average	Best
Dataset3	HC	86.53 ± 4.02	80.29 ± 12.86	80.63 ± 4.66	91.00 ± 10.25	82.79 ± 2.41	86.69 ± 8.30
	Acute-phase VKH	80.41 ± 9.18	96.00 ± 8.94	82.97 ± 2.49	83.41 ± 9.88		
	Convalescent-phase VKH	79.85 ± 5.76	86.95 ± 12.68	84.62 ± 3.57	86.36 ± 8.60		
Dataset4	HC	99.56 ± 1.15	100.0 ± 0.0	90.41 ± 2.31	93.56 ± 9.83	93.88 ± 1.37	96.16 ± 5.88
	Acute-phase VKH	94.55 ± 3.97	100.0 ± 0.0	93.82 ± 1.39	95.00 ± 7.45		
	Convalescent-phase VKH	88.26 ± 2.94	89.33 ± 15.35	97.55 ± 1.61	100.0 ± 0.0		
*P*	HC	<0.001	0.032	<0.001	0.690	<0.001	0.095
	Acute-phase VKH	<0.001	0.690	<0.001	0.095		
	Convalescent-phase VKH	<0.001	0.841	<0.001	0.032		

**FIGURE 4:**
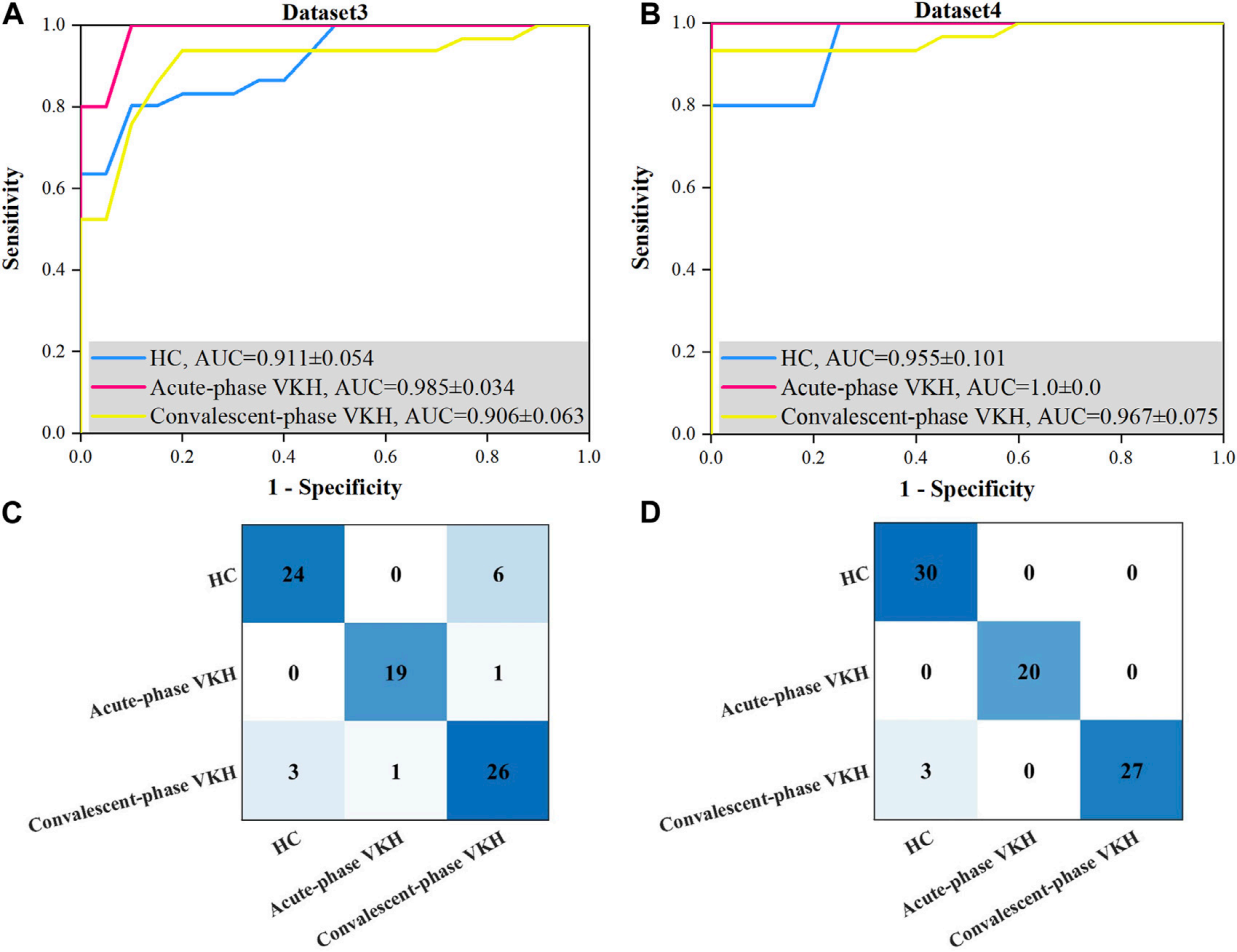
Best ROC curves and confusion matrices for three-class VKH classification with the proposed method based on Dataset3 and Dataset4. **(A)** Best ROC curve for Dataset3. **(B)** Best ROC curve for Dataset4. **(C)** Confusion matrix based on Dataset3. **(D)** Confusion matrix based on Dataset4.

Best sensitivity of the HC class derived for Dataset3 was poorer than its average sensitivity, which may be since the proposed method used the overall error for constructing the fitness function, and although the sensitivity of this class is lower, the sensitivity of the other two classes is much higher, which phenomenon leads to higher overall accuracy (lower overall error). In addition, the sensitivity and specificity of the other categories from Dataset 4 were excellent compared to those of Dataset3, but not significantly different. Although the best overall accuracy obtained using Dataset4 is not significantly better than that obtained based on Dataset3, the former has a smaller standard deviation. The AUCs of HC, acute-phase VKH, and convalescent-phase VKH classes acquired from Dataset4 (Figure 4B) were clearly outstanding than those expressed from Dataset3 (Figure 4A), although they were not significantly different (*p*-values of 0.310, 0.690, 0.310, respectively). As far as detailed misidentifications are concerned, the misidentifications of the classification model obtained with Dataset3 are concentrated between HC and convalescent-phase VKH, and sporadically between acute-phase and convalescent-phase VKH (Figure 4C), while in Dataset4 only three convalescent-phase VKH eyes were misidentified as HC eyes (Figure 4D). Regarding the selected features, the best feature subset obtained with Dataset3 included 13 features, specifically AFAZ, AI, peripapillary SVP-VLD, macular DVP-VLD, macular SVP-VPD, whole FOV SVP-VPD, peripapillary SVP-VPD, whole FOV DVP-VPD, peripheral CC-FVAR, macular CC-FV1000, peripheral CC-FV1000, macular CC-FVAS, and peripheral CC-FVAS, whereas the best feature subset retrieved from Dataset4 contains only 6 features, comprising logMAR BCVA, AFAZ, peripapillary SVP-VLD, macular SVP-VPD, macular DVP-VPD, and whole FOV CC-VPD.

### 3.4 Comparison of the proposed method with the previous VKH classification methods

The above results revealed that the scheme combining clinical characteristics and WSS-OCTA features could obtain better performance of two- and three-class VKH classification, so we took the performance obtained by this scheme as the final VKH classification performance. Given that this is the first study of VKH classification based on WSS-OCTA features and feature selection using SVM-EO to our knowledge, the classification performance was compared with previous methods for VKH classification based on other imaging features and machine learning. According to Table 4, the most outstanding VKH classification performance was achieved using WSS-OCTA features and the proposed SVM-EO method, both for distinguishing VKH and HC and for identifying acute VKH, convalescent VKH and HC, which demonstrated the effectiveness and superiority of the proposed method.

**TABLE 4:** Average and best three-class VKH classification results obtained by using the feature selection and classification method.

Paper	Classification	Sensitivity (%)	Specificity (%)	AUC	Accuracy (%)
Yang et al. (2018)	HC vs. VKH	94.6	92.2	0.934	-
Chen et al. (2020)	HC vs. active VKH	-	-	0.999	-
	HC vs. inactive VKH	-	-	0.902	-
Standardization of Uveitis Nomenclature SUN Working Group (2021a)	Early-stage VKH	-	-	-	92.3
	Late-stage VKH	-	-	-	88
Chang et al. (2021)	HC vs. VKH	-	-	0.808	-
	Active VKH vs. inactive VKH	-	-	0.958	-
This paper	HC vs. VKH	98.00 ± 2.00	100.0 ± 0.0	0.983 ± 0.039	98.82 ± 1.18
	Healthy control	100.0 ± 0.0	93.56 ± 4.39	0.955 ± 0.101	96.16 ± 2.63
	Acute-phase VKH	100.0 ± 0.0	95.00 ± 4.39	1.0 ± 0.0	
	Convalescent-phase VKH	89.33 ± 6.86	100.0 ± 0.0	0.967 ± 0.075	

### 3.5 Interpretable results for 2- and 3-class VKH classification models

**FIGURE 5:**
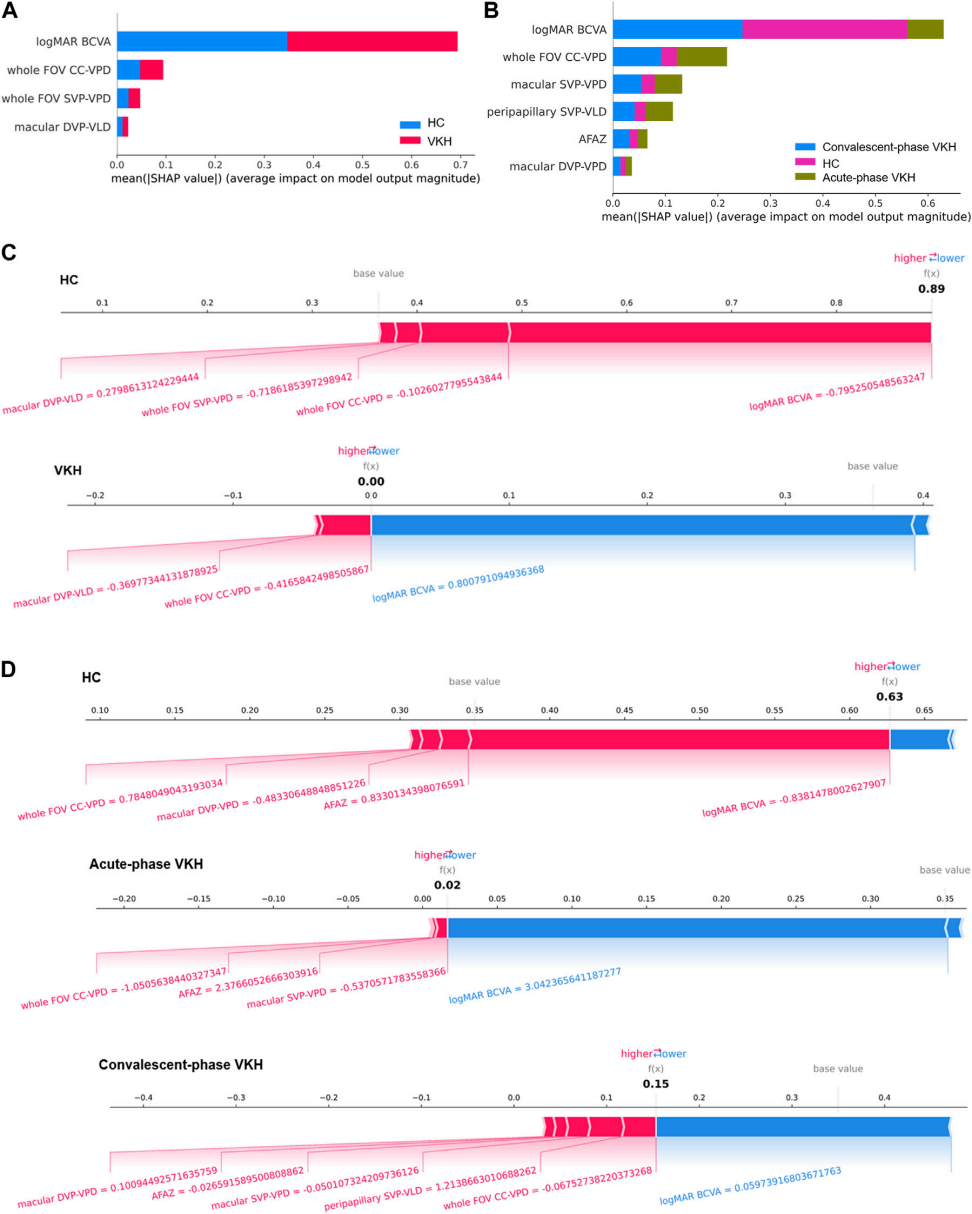
Global and local interpretability plots for SHAP-based 2- and 3-class VKH classification models. **(A–B)** Summary plots of feature importance for 2- and 3-class VKH classification models. **(C–D)** Local interpretability force plots for 2- and 3-class VKH classification models (The value of each feature is a standardized value).

The best features for the 2- and 3-class VKH classification have been given in the foregoing results, and the impact and contribution of these features to the VKH classification are presented here. Figures 5A, B revealed the contributions of each feature to the best 2- and 3-class VKH classification models. Overall, logMAR BCVA and whole FOV CC-VPD were the two most important features for the classification of VKH, whether two or three classifications. And the role of logMAR BCVA was far more important in both the 2-class task and the HC and convalescent-phase VKH categories in the 3- class task. Partially, the importance of each feature in each category may not be consistent with that of the overall classification task. For example, whole FOV CC-VPD was the most important feature for acute-phase VKH, not logMAR BCVA, the most important feature for three-class VKH classification. Figures 5C, D present some examples of the contributions made by each feature for the individual predictions from the best 2-class and 3-class VKH classification models. In the 2-class VKH classification, all features contributed to pushing up the HC class, while only macular DVP-VLD and whole FOV CC-VPD pushed up the VKH class, and logMAR BCVA had the largest effect in both the HC and VKH classes, but in the opposite direction. Similarly, logMAR BCVA had the largest effect in all classes in the 3-class VKH classification, but in the HC class with the acute-phase VKH and convalescent-phase VKH classes in the inverse direction. Whole FOV CC-VPD and AFAZ pushed up the predicted values in all classes, especially whole FOV CC-VPD also pushed up the HC and VKH classes in the 2-class VKH classification. These results confirmed the role of the selected optimal feature combinations in VKH classification, facilitating an interpretable and intelligent classification of VKH consistent with clinical diagnostic logic.

## 4 Discussion

In the past, many studies have focused on and confirmed microvascular changes in VKH disease based on OCTA (Uji et al., 2017; Karaca et al., 2020; Liang et al., 2020; 2021; Fan et al., 2021; Luo et al., 2021; Qian et al., 2021; Ye et al., 2021), however, no further OCTA-based classification models or criteria for VKH have been developed. In this paper, two two-class (VKH vs. HC) and three-class (HC vs. acute-phase VKH vs. convalescent-phase VKH) VKH datasets were established based on 2 clinical characteristics and 20 WSS-OCTA features including the FAZ, VPD, VLD, and FV features. The SVM-EO method was then used to simultaneously screen for VKH-sensitive features and optimize the hyperparameters of the SVM for effective VKH classification. To the best of our knowledge, this is the first VKH classification study based on WSS-OCTA features and with feature selection and classification by the SVM-EO method for enabling efficient performance. The results demonstrated.

That more efficient and stable classification performance of two- and three-class VKH with the combined clinical characteristics and WSS-OCTA features than with the pure WSS-OCTA features, and outperformed previous VKH classification studies.

Compared with previous studies, our proposed VKH classification method has differences and advantages both in terms of examination method and classification algorithm. In terms of the examination method, the current VKH classification relies on various imaging techniques or metabolomics. Previously, imaging techniques such as FA, ICGA, EDI-OCT, and SS-OCT were used extensively as auxiliary examinations and classifications for VKH (Hedayatfar et al., 2019; Urzua et al., 2020; Li et al., 2021). Based on clinical findings and these auxiliary examinations, several classification criteria for VKH have been proposed, such as RDC, the Classification Criteria for VKH Disease (DCV) (Yang et al., 2018), and the Standardization of Uveitis Nomenclature (SUN) criteria (Standardization of Uveitis Nomenclature SUN Working Group, 2021b). RDC overcomes the shortcomings of the Sugiura (Sugiura, 1978) and AUS criteria (Snyder and Tessler, 1980), and uses FA and ultrasonography findings as useful adjuncts, which is frequently used in clinical practice. The DCV takes into account unique clinical features and ancillary tests (OCT, B-scan ultrasonography, EDI-OCT, ICGA, and FA) and has achieved better AUC, negative predictive value, and sensitivity than RDC (Yang et al., 2018). The SUN criteria for VKH disease (fundus photographs, FA, and OCT) has many factors similar to DCV, but eliminates nonspecific exclusions with regionally relevant ones and does not include EDI-OCT findings (Standardization of Uveitis Nomenclature SUN Working Group, 2021a). In short, these criteria require complex clinical investigations and various imaging examinations, most of which are tedious, difficult to repeat, and invasive (e.g., ICGA and FA). Metabolomics-based analysis methods were also applied for VKH classification. Chen et al. (2020) used plasma metabolomics to identify significant differences in plasma metabolic phenotypes of VKH patients and identified diagnostic biomarkers for VKH disease. Chang et al. (2021) used urine metabolomics to identify predictive urine biomarkers for VKH disease. However, the VKH classification based on metabolomic analysis remains in laboratory research, and it is an expensive and time-consuming examination. Thus, a simplified and precise classification algorithm is needed and will be more conducive to VKH classification (Herbort et al., 2021). Using a simplified, rapid, and non-invasive imaging examination like WSS-OCTA with extracted disease-specific vascular parameters, along with common and easily measurable clinical characteristics (IOP and logMAR BCVA), we aim to develop a repeatable, easy-to-conduct VKH diagnostic method.

In addition, an advanced and efficient feature selection and classification method called SVM-EO was adopted to screen sensitive parameters and accurately classify VKH, which is the second particular advantage of this paper. Either imaging examinations combined with clinical characteristics or metabolomics leads to a large number of features, some of which are irrelevant, redundant and potentially reducing VKH classification performance, and therefore there is a necessity for feature selection of the extracted features. For example, Chang et al. (2021) identified 35 differential metabolites based on projection values obtained by principal component analysis (PCA) and orthogonal projection-discriminant analysis of potential structure (OPLS-DA) and statistical analysis and then modeled the classification of VKH by binary LR classifier. Yang et al. (2018) used latent class analysis (LCA) to screen 21 variables from 37 variables for the development of the best-fitting three-class VKH classification model. These methods are well-established and commonly used statistical analysis-based feature selection methods in medical practice, not machine learning-based methods, which may result in not great classification performance. Feature selection is essentially a global optimization problem, so metaheuristic algorithms are widely used for feature selection because of their powerful global search capability (Houssein et al., 2021b). The EO is a novel metaheuristic algorithm proven to outperform many classical optimization algorithms, and recent methods combining EO and machine learning algorithms such as KNN and SVM have been effectively applied to public datasets (Ouadfel and Abd Elaziz, 2022) and practical problems such as stock market prediction (Houssein et al., 2021a) and bearing fault identification (Tan et al., 2021). The classification performance of machine learning algorithms depends not only on the selected features but also on the optimality of their hyperparameters. However, the aforementioned studies either focus on feature selection or hyperparameter optimization of machine learning algorithms. In this paper, instead of feature selection or hyperparameter optimization alone, we integrated an EO and an SVM to simultaneously screen for the optimal features and hyperparameters to achieve the best VKH classification performance with the fewest features.

The classification results also demonstrate the feasibility and superiority of the proposed SVM-EO method. First, good VKH classification performance was achieved based on pure WSS-OCTA features, and the number of features was effectively reduced (5 and 13 features for 2- and 3-class VKH classification). Surprisingly, based on the combination of clinical characteristics and WSS-OCTA features, not only was the best combination of features with a smaller number of features (4 and 6 features for 2- and 3-class VKH) obtained, but also the significantly better average VKH classification performance and the best VKH classification performance with smaller standard deviations were obtained, both in terms of sensitivity, specificity, AUC, and accuracy (Tables 2, 3). Therefore, the combination of clinical characteristics and WSS-OCTA features was considered to be the optimal solution. Finally, the comparison with previous VKH classification studies found that the state-of-the-art VKH classification performance was accomplished based on this scheme and the SVM-EO method (Table 4). Notably, although Chen et al. (2020) achieved a higher AUC between HC and active VKH classes (0.999) than HC class (0.955), this paper achieved an AUC of 1 for the acute-phase VKH class in the 3-class VKH classification (Table 4) and no misidentification between the acute-phase VKH class and the HC class (Figure 4), indicating that this paper achieved better VKH classification performance.

In terms of the features screened, except for the AFAZ parameter (*p* = 0.055), the selected features were all parameters that proved to be significantly different between groups (Ye et al., 2021), indicating that the method of selecting sensitive parameters in this paper is reliable and valid. This also explains why FV parameters were not chosen for both 2- and 3-class VKH classification, as we previously found no significant differences in FV parameters between HC and convalescent-phase VKH (Ye et al., 2021). The VPD parameters accounted for half of the number of features screened for VKH classification, which also supported VPD as a sensitive indicator of VKH (Liang et al., 2020; Fan et al., 2021). Interestingly, only two of the screened features in the 2- and 3-class VKH classification (logMAR BCVA and whole FOV CC-VPD) were identical, the others were not. The reason for this phenomenon may be that some features, although easily distinguishable between HC and VKH, are difficult to distinguish between acute-phase VKH and convalescent-phase VKH, and *vice versa*. In addition, as the SVM classification model is a black box model (Chen and Gao, 2020), the global and local interpretability of the best 2- and 3-class classification models based on SHAP was investigated to discover the contribution of the selected features toward the VKH classification (Figure 5). Unlike previous studies that highlighted the correlation between OCTA features and logMAR BCVA (Liang et al., 2020; 2021; Qian et al., 2021; Ye et al., 2021), here Figure 5 exposes that the logMAR BCVA is the most important feature for VKH classification, which in turn justifies the impairment of visual acuity due to the onset and progression of VKH. However, the only logMAR BCVA is not sufficient for the classification of VKH, as it is not disease-specific, and many ophthalmic conditions such as diabetic retinopathy, retinal vein occlusion, and glaucoma can also cause visual changes and are correlated with the OCTA features of these diseases (Liang et al., 2021). The key contribution of WSS-OCTA features to VKH classification in the model is irreplaceable. On the one hand, these features are disease-specific. Both high 2- and 3-class VKH classifications were achieved based on WSS-OCTA features along with an accuracy of 91.62% and 86.69%. On the other hand, combining these features with logMAR BCVA not only enabled disease-specific classification but also achieved the best classification performance (98.82% and 96.16%), significantly outperforming them alone. In conclusion, WSS-OCTA features and clinical characteristics are compatible with each other and jointly contribute to highly specific and sensitive VKH classification.

There are some limitations in this paper. Firstly, the number of VKH patients and HC recruited was relatively low due to the strict subject inclusion and exclusion criteria, which resulted in a relatively small sample size. More VKH subjects will be recruited in our future work to increase the sample size. Moreover, including patients with other VKH stages will work for establishing a comprehensive diagnostic model for accurate grading of VKH phases. Secondly, although various WSS-OCTA features were screened, manually segmenting OCTA images and computing WSS-OCTA features were time-consuming and subjective, so automatically segmenting OCTA images and extracting WSS-OCTA features based on deep learning and image processing techniques will be carried out. Finally, conducting a multicenter prospective study of VKH intelligent classification will be helpful to validate and test the generalization and real-world classification performance of the VKH classification model established in this paper.

## 5 Conclusion

To establish a simplified and feasible VKH classification model, we carried out a VKH intelligent classification study based on WSS-OCTA images and clinical characteristics, and adopted a new feature selection and classification method named SVM-EO to improve the classification performance. The results showed that outstanding 2- and 3-class VKH classification performance was achieved. In addition, we investigated the interpretability of the VKH classification model based on SHAP, giving the importance ranking of the selected features and examples of the contribution of each feature to the prediction of a single sample, thus achieving VKH intelligent classification in accordance with clinical diagnostic logic. However, the modest sample size and artificially calculated WSS-OCTA features limit the clinical validation and application of this classification model. In the future, we will expand the sample size and adopt deep learning methods to achieve automatic segmentation and feature extraction of OCTA images for real-world applications of VKH intelligent classification.

## Data Availability

The original contributions presented in the study are included in the article/supplementary material, further inquiries can be directed to the corresponding authors.
